# Cyclopædia of Drug Pathogenesy

**Published:** 1887-11

**Authors:** 


THE CYCLOPÆDIA OF DRUG PATHOGENESY.—In the August number of this journal Dr. Taber published a criticism of the manner in which Lactic acid was given in the *Cyclopædia of Drug Pathogenesy.* A reply from Dr. Hughes is given in this issue. A sentence from this reply reads : "It is for those who wish to carry out Hahnemann's method (of prescribing) in its entirety, fitting drug action to disease on the principle *similia similibus*, that the *Cyclopædia* is constructed ; and I feel sure that they will appreciate its value." Hahnemann's method was to prescribe for symptoms and patients, not for *diseases ;* it is just there that Hahnemann and Dr. Hughes differ. Dr. Hughes would *condense* the materia medica so as to include only pathological results. The finer shades of symptoms are omitted ; it is these finer shades which enable us to individualize between remedies. The *Cyclopædia* omits them ; it omits all provings with potencies ; it is very badly arranged. Before the work was begun it was resolved and declared that no remedy should produce over five pages of "drug action," and that it should not be operative above a certain dilution ! Is this science or common sense ? The *Cyclopædia* was conceived and planned in this dogmatic, stupid matter, and the result is a *failure* which even Dr. Hughes' able pen cannot excuse.

